# Fibre-optic based exploration of lung cancer autofluorescence using spectral fluorescence lifetime

**DOI:** 10.1364/BOE.515609

**Published:** 2024-01-26

**Authors:** Alexandra C. Adams, András Kufcsák, Charles Lochenie, Mohsen Khadem, Ahsan R. Akram, Kevin Dhaliwal, Sohan Seth

**Affiliations:** 1 Translational Healthcare Technology Group, Institute for Regeneration and Repair, 5 Little France Dr, Edinburgh EH16 4UU, UK; 2 Institute of Photonics and Quantum Sciences, Heriot-Watt University, Edinburgh EH14 4AS, UK; 3 School of Informatics, University of Edinburgh, Edinburgh EH8 9AB, UK

## Abstract

Fibre-optic based time-resolved fluorescence spectroscopy (TRFS) is an advanced spectroscopy technique that generates sample-specific spectral-temporal signature, characterising variations in fluorescence in real-time. As such, it can be used to interrogate tissue autofluorescence. Recent advancements in TRFS technology, including the development of devices that simultaneously measure high-resolution spectral and temporal fluorescence, paired with novel analysis methods extracting information from these multidimensional measurements effectively, provide additional insight into the underlying autofluorescence features of a sample. This study demonstrates, using both simulated data and endogenous fluorophores measured bench-side, that the shape of the *spectral fluorescence lifetime*, or fluorescence lifetimes estimated over high-resolution spectral channels across a broad range, is influenced by the relative abundance of underlying fluorophores in mixed systems and their respective environment. This study, furthermore, explores the properties of the spectral fluorescence lifetime in paired lung tissue deemed either abnormal or normal by pathologists. We observe that, on average, the shape of the spectral fluorescence lifetime at multiple locations sampled on 14 abnormal lung tissue, compared to multiple locations sampled on the respective paired normal lung tissue, shows more variability; and, while not statistically significant, the average spectral fluorescence lifetime in abnormal tissue is consistently lower over every wavelength than the normal tissue.

## 1. Introduction

Lung cancer, the leading cause of cancer-related deaths worldwide [1], has a diagnostic deficit. Only 16.6% of patients are diagnosed with the disease at an early stage with an anticipated 5-year survival rate of 80% [2]. This drops to 10% for diagnoses made at an advanced stage. Thus, we need new diagnostic strategies that enable early and accurate diagnoses. The development of non-surgical radiation-free diagnostic alternatives, such as Fluorescence Lifetime Imaging Microscopy (FLIM) and Time-Resolved Fluorescence Spectroscopy (TRFS) have been shown to discriminate diseased tissue and, additionally, can be available bed-side [3–5]. When used in a label-free setting, these devices produce a detailed molecular profile of tissue autofluorescence (AF), potentially facilitating a more informed and rapid diagnosis [3,6].

**Fig. 1.**
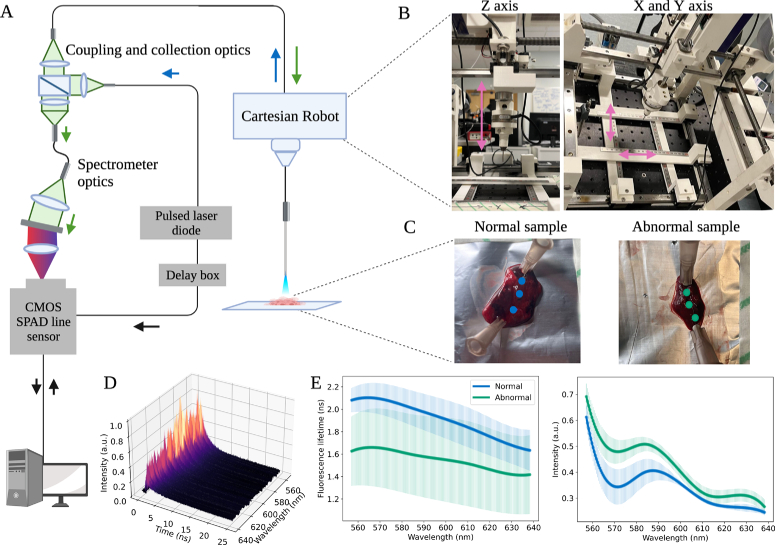
Illustration of the tissue data collection set up used in this study. **A)** The extensively parallel time-resolved fluorescence spectroscopy system used to collect high resolution spectral-temporal data across 512 wavelength channels across a wavelength range of 474.51 nm-735.12 nm with a resolution of 0.5 nm. The device has a pulsed laser with an excitation of 485 nm, coupling and collection optics, a spectrometer and a complementary metal-oxide semiconductor (CMOS) single photon avalanche diode (SPAD) line sensor. **B)** Cartesian robot is used to collect repeatable, accurate tissue data. The sensing fibre is mounted into the robot and moved across an X, Y and Z axis with 0.5 mm precision. **C)** Paired normal and abnormal, as decided by histopathology following lobe resection, samples were measured on the device. These samples were mounted on a collection board and pinned to prevent movement during the data collection process. **D)** An example of a preprocessed histogram of tissue data collected from the device. The device collects fluorescence data in the form of a histogram of fluorescence intensity over time and wavelength. **E)** An example of the different fluorescence profiles between a normal (blue) and abnormal (green) paired sample. Fluorescence intensity across the wavelength and the SFL of the samples were estimated using Multichannel Fluorescence Lifetime Estimation [26].

**Fig. 2.**
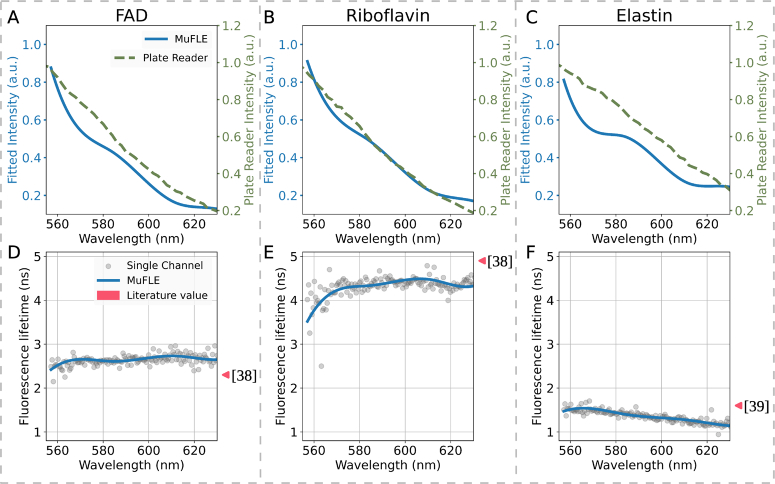
Spectral fluorescence profiles of 3 reference endogenous fluorophores: FAD **(A and D)**, Riboflavin **(B and E)** and Elastin **(C and F)**. (A), (B) and (C) show the fluorescence emission of the fluorophores measured on the EP-TRFS device modeled using MuFLE (blue) compared to the fluorescence emission measured on a plate reader (green dashed). (D), (E) and (F) show the spectral fluorescence lifetime of the fluorophores measured on the EP-TRFS device modeled using MuFLE (blue), compared to the single channel lifetime estimation (grey dots) modeled using the least squares method assessing the individual channels alone, and the values reported in the literature (pink triangles). The measurements were collected in a wavelength window between 557.13 nm-628.02 nm

AF describes the fluorescence of naturally occurring molecules, i.e., *endogenous fluorophores* [7]. Endogenous fluorophores, due to their intrinsic molecular structures, have distinct absorption and emission profiles when measured across a suitable wavelength range [8, Fig. 1] [9, Fig. 2]. In addition to the spectral emission, the average time the fluorophore spends in an excited state, known as the *fluorescence lifetime*, can be measured. This value is fluorophore specific and sensitive to the environment [7]. Therefore, in a given physico-chemical environment (i.e., with a set temperature, pH, viscosity, media dielectric constant, free or enzyme-bound), *a single fluorophore will exhibit a specific fluorescence lifetime and emission profile* [10].

Interpreting AF in lung tissue presents challenges arising from the overlapping excitation and emission profiles, similarities in lifetimes, and the unknown environments of endogenous fluorophores [10]. Depending on the optical setup, various devices measure the emission spectrum at distinct wavelength locations, also referred to as *channels* [11]. Each channel has a *bandwidth* (with smaller bandwidths implying higher *resolution*) within which photons are accumulated, collectively covering a specific wavelength *range*. Therefore, both the range and bandwidth dictate which fluorophores the device can measure, and, in the context of tissue delineation, this variability can lead to different conclusions (see Table 1 and Supplement 1). In addition, the systems previously employed for measuring AF collected fluorescence in a limited number of parallel spectral channels. For example, the ms-TRFS device [12], utilised in detecting oral and oropharyngeal cancer [13], assessed fluorescence across 4 spectral channels with resolutions ranging from 14 nm to 26.5 nm. In these setups, the underlying signal is essentially marginalised into distinct, low-resolution spectral bins, thereby limiting the detection of subtle yet potentially significant changes in the tissue being explored. Moreover, lung cancer is often characterised by structural deregulation [14], and consequently, alterations may manifest not only in the physical-chemical changes of lung AF but also in the relative abundance of distinct endogenous fluorophores. This insight could provide additional information about the variations in lifetime between normal and abnormal lung tissue.

**Table 1.** Reported lifetime changes in different cancer and normal samples.

Cancer type	Excitation wavelength	Emission channel	Lifetime change	Reference
Lung	485 nm	557.13 nm-638.22 nm	No significant difference	This paper
Lung	405 nm	510 nm-550 nm & 600 nm-640 nm	No significant difference	[15]
Lung	405 nm	430 nm	Decrease (by 0.55 ns)	[16]
Lung	488 nm	498 nm-570 nm	Decrease (by 0.32 ns)	[17]
Breast	780 nm & 890 nm	350 nm-720 nm, 16 channels, 10 nm wide	Increase (by 0.229 ns)	[18]
Breast	415 nm	No mention	Decrease (by 0.119 ns)	[19]
Cervical	405 nm	430 nm	Increase (by 1 ns)	[20]
Thyroid	298 nm-300 nm	340 nm & 450 nm	Increase (340 nm)	[21]
			No significant difference (450 nm)	
Colon	355 nm	375 nm	Increase (by 0.6 ns)	[22]
Gastrointestinal	355 nm	375 nm	Increase (by 0.44 ns)	[22]
Skin	435 nm	390 nm-600 nm, 16 channels	Decrease (by 0.620±0.340ns )	[23]

Development in TRFS technology has allowed for alternative devices which assess multichannel AF profiles across a large wavelength range and at a higher resolution (i.e., smaller bandwidth for individual channels) [11,24,25]. This allows for the interrogation of both the emission spectrum and *spectral fluorescence lifetime* (SFL), i.e., the variation of *average* fluorescence lifetimes in consecutive channels over a broad range. As discussed in [26] and [27, Chapter 3.2.1], given a single fluorophore, we expect SFL to be constant over wavelengths, but in the context of tissue, where multiple fluorophores are excited simultaneously, we expect SFL to vary, depending on the respective emission spectra of the underlying fluorophores and their relative prevalence in the sample (see section 3.1). The relative abundance of underlying fluorophores can also be estimated through fitting multiple exponentials [18] and spectral-temporal un-mixing. This approach, however, requires assuming that the number of underlying fluorophores are known *a priori*, and we consider SFL to be a proxy for this information. SFL may, therefore, provide additional discriminating information between a diseased and normal tissue sample, than when comparing the fluorescence over a single channel [28,29].

Previously, we reported on the extensively-parallel (EP-TRFS) device providing a high resolution fluorescence profile of a sample *over hundreds of channels* [26]. When used in time-correlated single-photon counting (TCSPC) mode, this device constructs a high-resolution histogram of fluorescence concurrently in the temporal and spectral domains. This device excites tissue at 485 nm. Notably, an excitation at 488 nm has been shown to differentiate lung cancer from normal tissue *in vivo* using an optical endomicroscopy (OEM) setup [30–32]. However, while OEM measures spatial fluorescence without a temporal assessment (thus restricting the fluorescence analysis to emission spectra), our approach evaluates lung AF through high-resolution spectral-temporal profiles.

In addition, a computational tool designed to interrogate the acquired high-resolution histograms, named Multichannel Fluorescence Lifetime Estimation or MuFLE, was suggested to provide detailed insights into fluorescence characteristics over multiple channels [26]. A FLIM setup with an excitation of 475 nm has also exhibited lung cancer discrimination using high-resolution spectral-temporal profiles, however, these lung profiles were assessed without a multi-channel analysis tool [24,33]. Therefore, using these recent developments, we assess the characteristics and applicability of SFL modeled from MuFLE in tissue delineation. Our assumption is that the relative abundances of endogenous fluorophores change in abnormal tissue compared to normal, due to the deregulation of tissue structure in neoplastic samples, thus, changing both the magnitude and shape of SFL, something which low resolution channel devices and conventional analysis tools are unable to capture.

Our overarching goal is to explore the utility of SFL, compared to alternative methods, i.e., *Aggregated Fluorescence Lifetime* (AFL) estimating fluorescence over marginalised histograms, in investigating mixed systems with multiple underlying fluorophores. Particularly in paired normal and abnormal lung tissue, e.g., in the context of delineating them. The contribution of the paper are as follows: first, we observe that the shape of the SFL is influenced by the relative abundance of underlying fluorophores when assessed in a mixed system. We show this to be true both when altering the relative abundance of simulated fluorophores and endogenous reference fluorophores while the physical-chemical environment (i.e., pH, temperature and viscosity of the solvent) remain unchanged, and *despite their emission spectra remaining similar* (see section. 3.1 and section. 3.2). Second, we show the shape of SFL, on average, shows more variability in abnormal tissue compared to normal tissue suggesting a more unpredictable alteration of fluorescence changes in suspected cancer tissue (see section. 3.3). However, we observe that there is considerable inter- and intra-sample heterogeneity between and within SFL of both normal and abnormal tissue in both magnitude and shape (see section. 3.3) limiting statistical significance where n=14 paired samples are investigated (i.e., 28 tissue sections from lobe resections or 90 spectral histograms in total). Third, we observe that although the AFL and SFL perform similarly in delineating lung tissue, the latter provides additional information that is not preserved when aggregating the fluorescence decays across all channels. To the best of our knowledge, this study is the first to explore the characteristics and utility of SFL in simulated data, bench-side fluorophores, and lung tissue.

## 2. Data and methods

### 2.1. EP-TRFS device

The EP-TRFS device is a fibre based setup which was used in time-correlated single photon counting (TCSPC) mode. In addition, the device was comprised of a pulsed laser (laser diode head (LDH-P-C-485, PicoQuant, Germany) and laser driver (PDL 800-D, PicoQuant, Germany) used at a repetition rate of 20 MHz with a complementary metal oxide semi-conductor (CMOS) single photon avalanche diode (SPAD) line sensor, as detailed in [26] (see Fig. 1 A). This allows high resolution histograms of photon arrival times measured at all channels in parallel using $1.5\times 10^{6}$ exposures, each with a 5μs exposure time and an optical laser output power of 175μW . Furthermore, we excite the sample such that we remain within the accepted photon detection rate of between 1 and 5% of laser pulses so as to mitigate pile-up risk [34]. Fluorescence spectroscopy measurements in both the temporal and spectral domain, across a broad spectral region between 474 nm-720 nm may be collected. The samples were excited at 485 nm, at this wavelength, as aforementioned, OEM devices have shown through exciting elastin, discrimination between normal and cancerous lungs are visible [30]. Due to the unique fibre-based setup, and spectral-temporal profile of the device, the instrument response function was measured using a quenched form of rose-bengal [35] (see section 4 for more details). Due to the narrower emission profile of rose-bengal, and the emission peaks of the endogenous fluorophores estimated to be excited by a laser of 485 nm, the total fluorescence spectral window the data was analysed in was between 557.13 nm-638.22 nm which consisted 160 individual spectral channels at a wavelength resolution of 0.5 nm.

### 2.2. Reference fluorophores

Three reference endogenous fluorophores: Flavin Adenine Dinucleotide (Sigma-Aldrich: F8384-100MG), Riboflavin (Sigma-Aldrich: R9504-25G) and Elastin (Sigma-Aldrich: E4527-1G) were measured on the EP-TRFS device. To standardise the environment of the fluorophores, the fluorophores were dissolved in _*dd*_H_2_O made up by the addition of buffer to a pH of 7. The fluorophores were collected at room temperature. Elastin was measured at a concentration of 500μM , FAD was measured at a concentration of 100μM and riboflavin was measured at a concentration of 100μM . To assess the fluorescence profile of mixed endogenous fluorophores in the same environment, 3 samples, referred to as mix 1, mix 2 and mix 3, containing the endogenous fluorophores at varying concentrations, at a pH of 7 were measured (see Supplement 1 Table. S1). To compare changes of SFL in different mixes, to that of different environments, mix 2 was made up in 2 more pHs, referred to as Mix 2a and Mix 2b. Mix 2a at a pH of 4 with the addition of 1 M hydrochloric acid, and Mix 2b at a pH of 9 following the addition of sodium hydroxide. To validate the intensity profiles and excitation wavelengths of all samples, the emission (see section. 3.2) was measured using the bottom read out of a plate reader (biotek, cytation 3 imaging reader).

### 2.3. Ex vivo lung tissue fragments

Tissue data from *ex vivo* lobectomy specimens, ranging from 50 mm^3^ cm in diameter were taken. Samples were obtained from patients undergoing lobe resections between January 2022 to January 2023, for suspected lung cancer (NHS Lothian BioResource, Scotland Research Ethics Service, reference 15/ES/0094). 14 lung samples, paired clinically as non-cancerous and cancerous, which we denote in the paper as normal and abnormal. Samples have been assessed and defined by type, stage and age (see Table 2). The pathological profile of the tissue samples vary. Depending on the size of the tissue sample, 3-6 locations per sample were assessed using our device, with a point sampling approach (fibre core diameter of 32μm ). At each location 3 repeated spectroscopy measurements were taken, with the same device setup as detailed in [26]. Data was collected using a 3 axis Cartesian robot [36] (see Fig. 1 B and C) to allow repeated measurements in the same X, Y and Z plane to be collected. The 3 repeated histograms were summed together before further assessment (see Fig. 1 D) to improve signal-to-noise ratio. Moreover, summed histograms with a peak intensity of below 300 were excluded from the analysis due to poor signal-to-noise.

**Table 2.** Sample information of the *ex vivo* lung tissue used in this study.

Sample label	Cancer type	Stage	Age	Sex
1	Adenocarcinoma	1B	63	F
2	Adenocarcinoma	1B	63	M
3	Adenocarcinoma	2B	73	F
4	Adenocarcinoma	1A	74	F
5	Adenocarcinoma	2A	77	M
6	Adenocarcinoma	3A	86	M
7	Adenocarcinoma	n/a	83	F
8	Squamous cell	3A	77	M
9	Squamous cell	3A	66	M
10	Squamous cell	3A	67	M
11	Squamous cell	3A		F
12	Squamous cell	2B	77	F
13	Large cell neuroendocrine	3A	81	M
14	Malignant melanoma	n/a	83	M

### 2.4. Analysis

The data from the EP-TRFS device was analysed using two different approaches.

#### 2.4.1. Aggregated fluorescence lifetime

To compare signal measured in the EP-TRFS device with signal from systems measuring autofluorescence in the more traditional low resolution channel method, the 160 channels histogram was aggregated into two distinct channels of 40 nm each (see Supplement 1 sec. 1.B and supplementary Fig. S1), and single lifetime, referred to as AFL, was estimated using least squared fitting. The single exponential fluorescence decay is modelled as follows:

(1) $$s[m]=(f*h)[m]+b\text{ and }f[m]=\gamma \text{exp}(-t_{m}/\tau ).$$

where γ and τ are the average intensity and fluorescence lifetime, b is the bias due to the dark counts of the detector and the fibre background, h[m] is the IRF at the channel, and ∗ denotes convolution. Given y[m] as the observed signal at the m-th bin, γ and τ can be estimated by minimising the loss function

(2) $$J_{1}=\sum_{m=1}^{M}{\left(y[m]-s[m]\right)}^{2}$$

assuming h[m] is known.

#### 2.4.2. Spectral fluorescence lifetime

In the second approach, each channel is analysed either separately with least squares fitting or simultaneously with MuFLE. Given multiple channels, MuFLE, simultaneously estimates the fluorescence intensity and lifetimes over these channels [26]. Here, the fluorescence decay is modelled as

(3) $$\begin{array}{ll} s[p,m] & =(f[p]*h[p])[m]+b[p]\text{ and }\\ f[p,m] & =\gamma [m]\text{exp}(-t_{m}/\tau [m]) \end{array}$$

where γ[p] and τ[p] are spectral intensity and lifetime over channels, and h[p][m] is the IRF at the p-th channel. In MuFLE, both γ[p] and τ[p] are modelled using B-splines basis functions ( $B_{i}$ ) as described in [26]. We find the optimal coefficients ( $a_{i}$ ) for the emission spectrum and SFL given $\gamma (\omega )=\sum_{i=1}^{I_{\gamma }}a_{i}^{\gamma }B_{i}(\omega )$ and $\tau (\omega )=\sum_{i=1}^{I_{\tau }}a_{i}^{\tau }B_{i}(\omega )$ . Following [26], we use cubic splines with 3 equidistant internal knots to generate the spline basis functions. Given y[p,m] as the observed signal at the m-th bin and p-th channel, the spline coefficients can be estimated by minimising the loss function

(4) $$J_{2}=\sum_{p=1}^{P}\sum_{m=1}^{M}{\left(y[p,m]-s[p,m]\right)}^{2}$$

assuming h[p,m] is known.

#### 2.4.3. Simulated data

Simulated data was generated to validate the hypothesis that SFL is sensitive to variation in the relative prevalence of fluorophores, even if the emission intensities are very similar to each other, i.e., without distinct peaks. A similar situation arises in the EP-TRFS system and lung tissue since the selected emission wavelength range only captures the tail end of emission spectra of the fluorophores of interest (see Fig. 2). Three hypothetical fluorophores were simulated using the model described in Eq. (3) with different intensity and lifetimes. Exponential and hyperbolic functions were used to model the emission intensities, i.e., γ[p] , of the individual fluorophores. These functions were chosen to replicate the tail of the emission profile of real fluorophores excited at 485 nm. Across the wavelength range, the lifetime was simulated to be fixed for each fluorophore, i.e., τ[m]=τ .

The temporal resolution was set to 0.05 ns to match the real data and a total of M=490 time bins were simulated. The number of channels was set to P=160 to match the real data. To simulate mixed samples, the histograms of the individual fluorophores were summed together and convolved with a simulated IRF. The simulated IRF was assumed to be exponential with a lifetime of 0.378 ns, matching the average lifetime of the IRF when fitted with a single exponential decay, recorded in the EP-TRFS device. The intensity at each channel and bin were perturbed using a Poisson noise.

## 3. Results

### 3.1. Simulation

It is expected that both the shape and magnitude of the SFL profile is influenced by the relative concentration of the underlying fluorophores with different but constant lifetimes. In the simplest model, we can consider a multi-exponential decay ( y ) of two fluorophores, in the same environment, a and b as

(5) $$y(\omega ,t)=\gamma_{a}(\omega )\text{exp}(-t/\tau_{a})+\gamma_{b}(\omega )\text{exp}(-t/\tau_{b})$$

where ω represents the wavelength, t represents time, and τ represents lifetime. As the intensities, reflecting the individual emission spectrum of the individual fluorophores, change across the wavelengths, the *average* lifetime, when approximated by a single exponential will also vary over the wavelengths. When $\gamma_{a}(\omega )<\gamma_{b}(\omega )$ , the average lifetime will be closer to $\tau_{b}$ , and when $\gamma_{a}(\omega )>\gamma_{b}(\omega )$ , the average lifetime will be closer to $\tau_{a}$ .

**Fig. 3.**
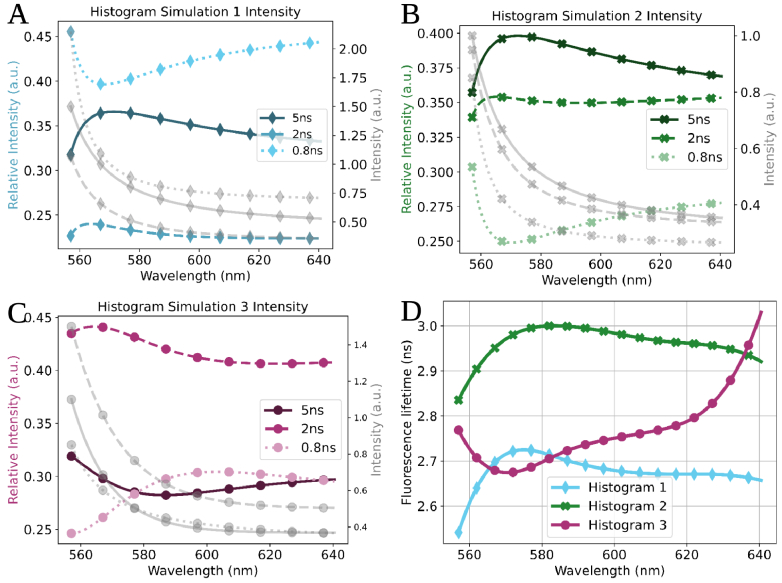
The relative intensity (coloured), individual intensity (grey) and estimated single exponential lifetime MuFLE results from simulated histograms. **A), B) and C)** represent the underlying relative intensity and emission spectra of 3 individual fluorophores which make up the final observed histograms in the simulated data. **D)** represents the single exponential estimated spectral fluorescence lifetime using the MuFLE model of the 3 different simulated histograms.

The 3 individual simulated fluorophores had similar spectral intensity profiles to that of the endogenous fluorophores used, i.e., they drop monotonically at longer wavelengths (see section 2.4.3). In addition, they also had similar fixed lifetime values to that of the endogenous fluorophores. We observe that the shape of SFL reflects the relative abundance of fluorophores. For example, a dip is observed in the SFL in histogram 3 just before 580 nm (see Fig. 3 D), this aligns with the wavelength range where the fluorophore corresponding to the highest lifetime is declining (see Fig. 3 C), in addition, it aligns with the wavelength range where the fluorophore with the lifetime of 2 ns has started to decline, and the fluorophore with a lifetime of 0.8 ns has started to rise (see Fig. 3 C). In both simulated histograms 1 and 2, a similar trend in the SFL shape is also observed that reflects the relative abundance of the fluorophores (see Fig. 3). In particular, the peaks seen in both SFL lifetime ranges between 560 nm-580 nm, reflect the decline in relative abundance of the fluorophore with a lifetime of 0.8 ns and increase in relative abundance of both fluorophores with a lifetime of 5 ns and 2 ns.

### 3.2. Endogenous fluorophores

We replicate the same experiment in the simulated data using three endogenous fluorophores bench-side. We select three endogenous fluorophores known to be excited at 485 nm in lung tissue, and are therefore also likely to be present in the tissue samples. We first validated the absorption (to confirm the excitation at 485 nm) and emission spectra of 3 endogenous fluorophores (see Fig. 2). The emission profiles from a plate reader were in agreement with the single exponential MuFLE intensity results of the individual fluorophores (see Fig. 2 A, B, C). The magnitude of SFL estimated using MuFLE was also in agreement with the *ground truth* values reported in the literature while the shape remained broadly constant [37,38] (see Fig. 2 D, E, F).

**Fig. 4.**
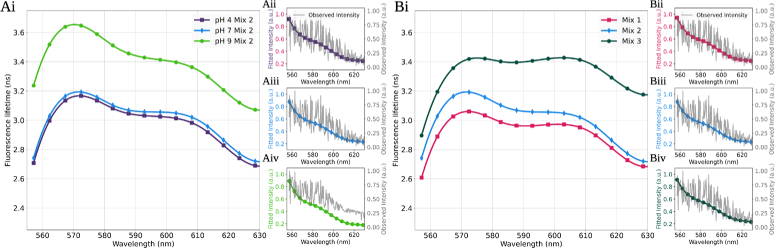
**A)** shows spectral fluorescence profiles of 3 samples which contain the same concentration of reference endogenous fluorophores: FAD, Riboflavin and Elastin, however, the pH of the solvent has been altered changing the environment of the fluorophores. **B)** shows spectral fluorescence profiles of 3 different mixes of the reference endogenous fluorophores: FAD, Riboflavin and Elastin all measured in a pH of 7 at room temperature. Mix 1 (pink squares) is comprised of 500μM of elastin, 50μM of riboflavin and 100μM of FAD. Mix 2 (blue diamonds) is comprised of 500μM of elastin, 50μM of riboflavin and 50μM of FAD. Mix 3 (green dots) is comprised of 400μM of elastin, 100μM of riboflavin and 100μM of FAD. **i** shows the spectral fluorescence lifetime estimated using the MuFLE model, of the 6 different mixed samples. **ii), iii) and iv)** show the emission profile of the mixed solutions, estimated using the MuFLE model, compared to the observed intensity when measured from the EP-TRFS device (grey line). The measurements were collected in a wavelength window between 557.13 nm-628.02 nm.

Following the validation, the mix of these fluorophores were analysed (see section 2.2 and Supplement 1). In all cases, similar intensity profiles of the samples were observed (see Fig. 4 ii-iv). When the environment of the sample was altered, a significant increase in the absolute lifetime value (average of 3.38ns±0.18 ) of Mix 2b (Mix 2 made up to a pH of 9 as mentioned in section. 2.2) was observed, compared to when measured in Mix 2 or Mix 2a (Mix 2 made up to a pH of 4 as mentioned in section. 2.2) (with an average lifetime value of 2.99ns±0.15 and 2.95ns±0.15 respectively) (see Fig. 4 Ai). When the relative concentration of the individual fluorophores was altered, independent of the environment (see Supplement 1), both the absolute value of the fluorescence lifetime (average lifetime: Mix 1: 2.89ns±0.12 , Mix 2: 2.99ns±0.15 and mix 3: 3.33ns±0.11 ), and the spectral fluorescence shape of the lifetime across the wavelength changed (see Fig. 4 Bi). The most notable spectral shape change was observed in Mix 3 which reached a plateau across the wavelength between 570 nm-610 nm, instead of a decline in value. The higher lifetime of mix 3 is expected from higher abundance of riboflavin and lower abundance of elastin compared to mix 1 and 2, while the slight lower lifetime of mix 1 compared to mix 2 is expected due to higher abundance of FAD.

### 3.3. *Ex vivo* lung tissue autofluorescence

The tissue samples were analysed both in terms of AFL and SFL. We assessed their characteristics both in normal and abnormal tissue samples in individual patients separately (indv), and normal and abnormal tissue samples pooled together across all individuals (pool).

#### 3.3.1. AFL:

##### 3.3.1.1 pool

Despite no significant difference, a slight decline is observed between the average lifetime of both channels from all normal samples (shorter wavelength channel: average lifetime of 1.49ns±0.47 , longer wavelength channel: average lifetime of 1.35ns±0.41 ) compared to the abnormal samples (shorter wavelength channel: average 1.37ns±0.48 , longer wavelength channel: average of 1.30ns±0.40 ) (see Supplement 1).

##### 3.3.1.2 indv

**Fig. 5.**
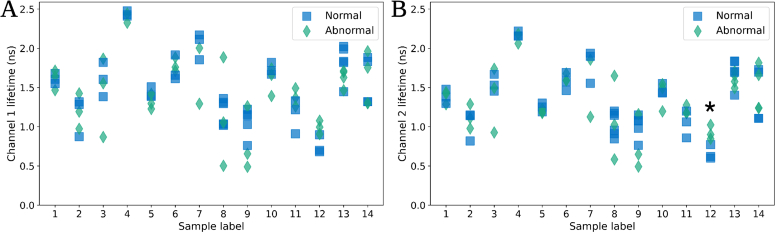
The fluorescence lifetime, estimated using the least squares method, of all paired normal (blue square) and abnormal (green diamond) samples are shown. **A)** shows the lifetime measured from the first spectral channel (shorter wavelength channel: 557.13 nm-597.42 nm). **B)** shows the fluorescence lifetime measured from the second spectral (longer wavelength channel: 597.93 nm-638.22 nm). * denotes statistical significance, i.e., p-value of t-test is less than 0.05.

In the shorter wavelength channel (see Fig. 5 A) no significant difference was observed between any paired sample. In the longer wavelength channel (see Fig. 5 B), a significantly higher lifetime value of the abnormal lifetime compared to the normal lifetime between one sample (sample number 12, p-value 0.03) was observed.

#### 3.3.2. SFL: magnitude

##### 3.3.2.1 pool

**Fig. 6.**
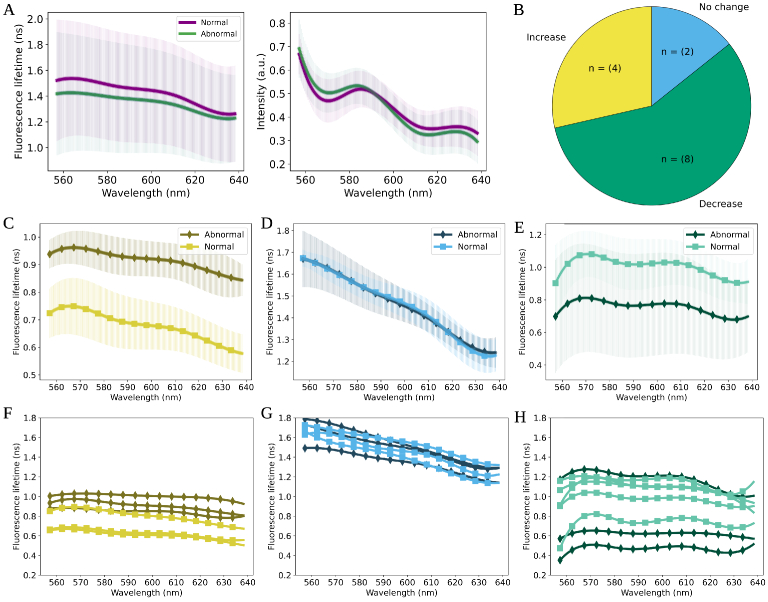
Differences in the fluorescence profiles between paired normal and abnormal samples are shown. **A)** shows the average spectral profiles of all tissue samples labelled by histology as normal and abnormal. The left plot shows an overall decline in the average SFL of abnormal samples (green) compared to the paired normal samples (purple). The figure also shows a large amount of variance between the average normal and abnormal values. The right plot shows the average fluorescence intensity captured between the normal (purple) and abnormal (green). All tissue data has been analysed across the wavelength profile of between 557.13 nm-638.22 nm. **B)** shows a pie chart representing the proportion of all 14 samples where an increase, decrease or no change between the normal and the abnormal SFL was observed. **C)** shows the SFL between the sample that has the largest increase in lifetime in the abnormal tissue, compared to the normal tissue (sample label: 12). **D)** shows the SFL between the sample where no change between the normal and abnormal tissue was observed (sample label: 1). **E)** shows the SFL between the sample where the greatest decrease in the SFL was observed in the abnormal tissue compared to the normal paired sample (sample label: 9). **F), G) and H)** shows the absolute lifetime values of the different locations of the paired normal and abnormal samples from the above plots.

A consistent decrease in the average SFL is observed in abnormal samples compared to normal samples at each channel (see Fig. 6 A). A t-test at each channel between pooled normal and pooled abnormal samples reveal that the drop in lifetime is not significant. When considering the average SFL of all samples, the most prominent difference is observed in the lower wavelength region (between 560 nm - 580 nm) (see Fig. 6 A). Furthermore, a declining trend in the value of lifetime across the wavelength range is observed, independent of sample type (see Fig. 6 A). These observations align with some reported in the literature, i.e. [23].

##### 3.3.2.2 indv

To assess sample specific SFL changes, first, a paired t-test (paired at 160 channels) between the average normal and abnormal SFL was conducted. In 8 of the samples, a consistent decrease in fluorescence lifetime between the average normal and average abnormal sample was observed. In 4 of the samples a consistent increase was observed, and in 2 no significant change was observed, however, in these samples abnormal SFL is higher than normal SFL towards longer wavelengths (see Fig. 6 B). To present some sample specific SFL, three samples were visualised (see Fig. 6 C, D and E). These samples had the largest increase, largest decrease and no significant change between the SFL.

In all samples, overlap in the absolute lifetime value in at least one tissue location is observed (see Supplement 1). Furthermore, the overlap in the lifetime values does not appear constant, with some wavelength ranges having a greater amount of overlap (i.e. in Fig. 6 F and in Fig. 6 H, one location overlaps in the lower wavelength range (560 nm - 570 nm) more prominently). Furthermore, in the sample which shows the greatest decrease in SFL (see Fig. 6 E and H), two of the abnormal locations appear lower than the normal range of between 1.2 ns and 0.7 ns, however, one of the abnormal locations appears in the higher region of the normal locations. T-tests assessed at each channel did not reveal significant change in lifetime except sample 12 where a p-value of less than 0.05 was observed in most wavelength ranges as also demonstrated by AFL.

#### 3.3.3. SFL: shape

##### 3.3.3.1 pool:

To assess the SFL shape, independent of the absolute lifetime value, the Procrustes disparity between all samples was calculated. The median disparity between all normal SFL was 0.076, whereas the median disparity between all abnormal SFL was 0.159, suggesting a larger variety within the SFL shape in the abnormal than the normal. The pairwise distances (measured using the Procrustes distance metric) between all samples was assessed and plotted using tSNE dimensionality reduction (see Supplement 1). A slight difference between sample type can be observed (see Supplement 1), where, irrespective of the sample being normal or abnormal, the squamous cell samples and adenocarcinoma samples separate (see Supplement 1).

##### 3.3.3.2 indv:

**Fig. 7.**
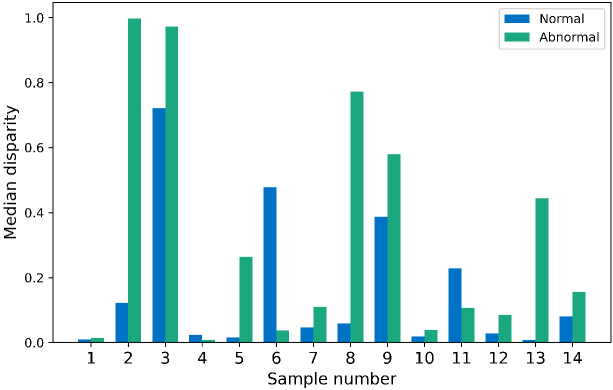
Median disparity calculated from a Procrustes similarity test between the normal (blue) and abnormal (green), paired spectral fluorescence lifetime measurements of 14 paired, clinically defined normal and abnormal *ex vivo* lung cancer samples.

The median disparity between the sample specific normal and abnormal SFL was assessed (see Fig. 7). Two samples had noticeably higher median disparity within the normal, compared to the abnormal. However, the other samples either had a combined negligible median disparity suggesting very similar shapes (i.e. less than 0.1), or had a markedly higher disparity within the abnormal sample, compared to the normal sample. Most notable in samples number 2 and 8 which had a median disparity of 0.99 and 0.77 in their abnormal, compared to 0.122 and 0.058 in their normal (see Fig. 7).

## 4. Discussion

Previous systems used to measure tissue AF are limited in collecting fluorescence in low resolution spectral channels (i.e., [12]). Innovations into both high-resolution spectral devices, and analysis techniques to interrogate these signals allows the total temporal spectral histogram to be assessed in detail. The EP-TRFS device and MuFLE algorithm enable high resolution fluorescence intensity and SFL estimation spanning across an entire emission spectrum from 557.13 nm-638.22 nm providing unforeseen details of tissue AF [26].

We demonstrate that the magnitude and shape of SFL is influenced by the relative abundance of underlying fluorophores and their respective lifetimes (see Fig. 4). We observe this to occur while changing the relative abundance of both simulated fluorophores and endogenous fluorophores in samples matched to the expected fluorophores emitting in tissue with the same optical setup. We also observe that while changing the environment of the endogenous fluorophores, the SFL shape remains relatively similar. This provides a potential explanation to the variation we observe in the SFL tissue data where the magnitude and shape vary considerably. We might expect the magnitude and shape of SFL to not change if the relative abundance of the underlying fluorophores and their environment remain homogeneous e.g., in healthy, organised tissue. We might expect the magnitude to change, while shape remaining similar, if the lifetime of individual fluorophores changes without the respective emission spectra changing, and we might expect both magnitude and shape to vary if the relative abundance of the fluorophores change while the respective lifetimes remain fixed, or if both the relative abundance and lifetimes change, e.g., in heterogeneous, disorganised abnormal tissue, like cancer. We show that aggregating the spectral channels into channels with large bandwidths prevents these subtle spectral information between normal and abnormal tissue to be assessed. Although these observations align with our expectation and our results broadly align with results reported in existing studies, a complete validation of the hypothesis is beyond the scope of this paper. Nonetheless, we observe SFL to provide a detailed overview of tissue characteristics, which may provide potential for improved and informed diagnostics.

A limitation to the EP-TRFS device used in this study is that it produces fluorescence profiles alone. Therefore, specific structures observed within the sample which may account for a unique fluorescence profile, such as a fibrotic area, are not recorded alongside the fluorescence profile. So, without direct microscopic images of structure, intra-heterogenous data will arise from mixed tissue components. Furthermore, inter-heterogeneity may arise from differences in patient history i.e. exposure to smoking, pollution and other environmental factors which may alter the inflammatory state of the lung, limiting how normal the normal counterpart is. Additional limitations of our study include the limited patient sample numbers, and the assumption that every location of a tissue sample we take measurements from is biologically the same. Therefore, the lack of significance can be attributed to both the smaller number of patients included in the study and smaller number of locations sampled for each tissue sample (resulting in a smaller number of point-based histogram samples accumulated per tissue area being assessed) as well as patient demographics and tissue environment.

Although the SFL magnitude and AFL do not show statistical significance in our analysis, the SFL shape show significant variability in abnormal (and often normal) lung tissue indicating the underlying complex molecular dynamics of the structure. Although the significance of the statistical test can be improved through additional data (assuming the difference of lifetime being 50 ps and the population standard deviation being 200 ps, 253 samples will lead to a significance test with level 0.05 and power 0.8), the underlying heterogeneity should be understood in more detail. E.g., if it is due to underlying physical-chemical processes that can both contribute to increase and decrease of lifetime [16], or due to patient demographics such as age or potentially smoking status, or due to the locality of the abnormality, e.g., if it is smaller than the field of view (i.e., <32μm ). A better understanding will lead to more informed diagnosis in precision medicine with SFL providing more granular information than AFL.

A major limitation within the spectral range utilised in the analysis, due to the IRF measured, remains. Since we use a fibre-based EP-TRFS system, and our device contains bandpass filters, traditional methods for measuring the IRF remain ineffective. For example, shining light directly onto the sensor does not account for the time delay of the optical signal travelling back and forth through the fibre. Similarly, reflecting excitation light back through the fibre is not appropriate since a bandpass filter blocks light less than 520 nm from entering the detector. A proper alignment of IRF and the observed spectra remain crucial for the least squares fitting routine, and therefore, to measure the IRF, we opt for using a fluorophore that can be excited at 485 nm with as wide a spectral range as possible and with a decay rate shorter than the pulsed light source. Quenched Rose-Bengal has been used in this manner [35]. Variations of fluorescein sodium was also tested (data not shown) at various concentrations, however, we found that the decay rate of this modified fluorophore remained wider than the laser pulse.

An excitation of 485 nm in previous studies using OEM systems has been shown to discriminate normal from abnormal structures, therefore, as aforementioned, the same setup was applied with this method. However, the excitation of the specific endogenous fluorophores with this device, coupled with the IRF limitation prevent the emission peaks of either fluorophore to be measured. Therefore, the optical setup misses the emission peak of the endogenous fluorophores which are expected to be excited using a 485 nm excitation wavelength. However, we show, using both simulated data and data generated bench-side that, regardless of the presence of an emission peak, the ratio of the individual fluorescence compartments influence the SFL shape. A potential discrimination enhancement of this application is to alter the device setup. More specifically, to investigate this emission at a shorter excitation wavelength, i.e., 350 nm, so as to increase the amount of endogenous fluorophores being excited. However, this is beyond the scope of this paper.

## 5. Conclusion

Our study demonstrates that the relative concentration of individual fluorophores contributes significantly to the shape and magnitude of SFL, both in simulated data and reference data while this information is not visible in AFL. This emphasises the importance of utilising high-resolution spectral channel data when evaluating tissue AF. Specifically focusing on lung tissue, we observed that, in most cases, the SFL consistently drops in magnitude across all channels in abnormal tissue compared to normal *ex vivo* lung tissue, although this difference is not statistically significant. Additionally, we noted that the shape of the SFL, on average, displays greater variability in abnormal *ex vivo* lung tissue compared to normal tissue. Furthermore, our observations revealed significant inter- and intra-heterogeneity among the patient samples measured. While this limits the immediate translation of this approach to tissue delineation, SFL provides unprecedented details on the tissue AF that can help better investigate the underlying tissue heterogeneity.

## Data Availability

Data presented here is available upon request.

## Supplemental document

See Supplement 1 for supporting content.
