# Supplementary material for: Fibre-optic based exploration of lung cancer autofluorescence using spectral fluorescence lifetime
Source: Biomed Opt Express. 2024 Jan 26;15(2):1132–47. doi: 10.1364/BOE.515609 (PMC10890895; doi:10.1364/BOE.515609)
Supplement: Supplementary file 1 [file boe-15-2-1132-s001.pdf]

Supplemental Document

Biomedical Optics EXPRESS

Fibre-optic based exploration of lung cancer autofluorescence using spectral fluorescence lifetime: supplement

ALEXANDRA C. ADAMS,[1] ANDRÁS KUFCSÁK,[2] CHARLES LOCHENIE,[1] MOHSEN KHADEM,[1,3] AHSAN R. AKRAM,[1] KEVIN DHALIWAL,[1] AND SOHAN SETH[1,3,*]

[1] *Translational Healthcare Technology Group, Institute for Regeneration and Repair, 5 Little France Dr, Edinburgh EH16 4UU, UK*
[2] *Institute of Photonics and Quantum Sciences, Heriot-Watt University, Edinburgh EH14 4AS, UK*
[3] *School of Informatics, University of Edinburgh, Edinburgh EH8 9AB, UK*
[*] *sohan.seth@ed.ac.uk*

This supplement published with Optica Publishing Group on 26 January 2024 by The Authors under the terms of the Creative Commons Attribution 4.0 License in the format provided by the authors and unedited. Further distribution of this work must maintain attribution to the author(s) and the published article's title, journal citation, and DOI.

Supplement DOI: https://doi.org/10.6084/m9.figshare.24972720

Parent Article DOI: https://doi.org/10.1364/BOE.515609

Fibre-optic based Exploration of Lung Cancer Autofluorescence using Spectral Fluorescence Lifetime: supplemental document

1. SUPPLEMENTARY MATERIAL

A. Endogenous Fluorophore Mixes

Table S1. Endogenous fluorophore mixes used in this study.

	Fluorophore concentration (uM)		
Mix Number	FAD	Riboflavin	Elastin
1	100	50	500
2	50	50	500
3	100	100	400

Table S2. Lifetime value of normal and abnormal samples recorded on difference devices.

Cancer type	Excitation wavelength	Emission channel	Lifetime change	Device temporal resolution	Device channel width	Tissue preparation	Reference
Lung	485 nm	557.13 nm-638.22 nm	No significant difference	50 ps	0.5 nm	*ex vivo*	Current paper
Lung	405 nm	510 nm-550 nm & 600 nm-640 nm	No significant difference	No mention	40 nm	*in vivo*	[1]
Lung	405 nm	465 nm-495 nm & 545 nm-580 nm	Decrease (by 0.55 ns)	No mention	30 nm & 35 nm	Paraffin-embedded	[2]
Lung	488 nm	498 nm-570 nm	Decrease (by 0.36 ns)	1.6 ns	72 nm	*ex vivo*	[3]
Breast	780 nm & 890 nm	350 nm-720 nm<br>16 channels	Increase (by 229 ps)	0.2 ns	10 nm	Mouse model<br>of human breast cancer<br>fixed Paraffin-embedded	[4]
Breast	415 nm	No mention	Decrease (by 119 ps)	0.4 ns	No mention	paraffin wax by an alcohol<br>only process	[5]
Cervical	405 nm	Above 430 nm,<br>No mention of precise channel	Increase (by 1 ns)	No mention	No mention	fixed and paraffin-embedded	[6]
Thyroid	298 nm-<br>300 nm	340 nm & 450 nm	Increase (340 nm)<br>No significant difference (450 nm)	0.012 ns per channel	No mention	*ex-vivo*, untreated	[7]
Colon	355 nm	375 nm	Increase (by 0.6 ns)	0.25 ns	No mention	formaldehyde fixation	[8]
Gastrointestinal	355 nm	375 nm	Increase (by 0.44 ns)	0.25 ns	No mention	formaldehyde fixation	[8]
Skin	435 nm	390 nm-600 nm 16 channels	Decrease (by 620 ps±340)	No mention	10 nm	*ex vivo* - untreated	[9]

Table S3. Average channel lifetime values, T statistic and P value results.

Sample label	Normal Shorter wavelength channel lifetime (ns)	Abnormal Shorter wavelength channel lifetime (ns)	Shorter wavelength channel p value	Normal Longer wavelength channel lifetime (ns)	Abnormal Longer wavelength channel lifetime (ns)	Longer wavelength channel p value
1	1.61	1.59	0.78	1.38	1.38	0.98
2	1.16	1.29	0.48	1.04	1.13	0.56
3	1.6	1.87	0.3	1.55	1.39	0.57
4	2.44	2.02	0.23	2.18	2.08	0.21
5	1.43	1.35	0.2	1.25	1.20	0.25
6	1.73	1.67	0.64	1.58	1.62	0.60
7	2.05	1.59	0.46	1.80	1.49	0.55
8	1.17	1.07	0.74	1.04	1.02	0.95
9	1.06	0.78	0.36	1.01	0.77	0.35
10	1.73	1.23	0.24	1.47	1.41	0.63
11	1.15	1.37	0.21	1.03	1.2	0.22
12	0.76	1.19	0.21	0.66	0.92	0.03
13	1.82	1.33	0.06	1.69	1.46	0.22
14	1.69	1.46	0.66	1.51	1.57	0.83

B. Marginalised Histogram

To compare high-resolution spectral lifetime against alternative methods from the literature, the histogram was aggregated into two distinct channels. This involved summing 80 fluorescence decays across two different wavelength ranges, mimicking two broad spectral channels: one between 557.13 nm and 597.42 nm, which we label shorter wavelength channel, and the other between 597.93 nm and 638.22 nm, which we label longer wavelength channel. In addition, the IRF recorded across the histogram, as mentioned above, was aggregated in the same way (see supplementary Fig. S1).

2. SUPPLEMENTARY FIGURES

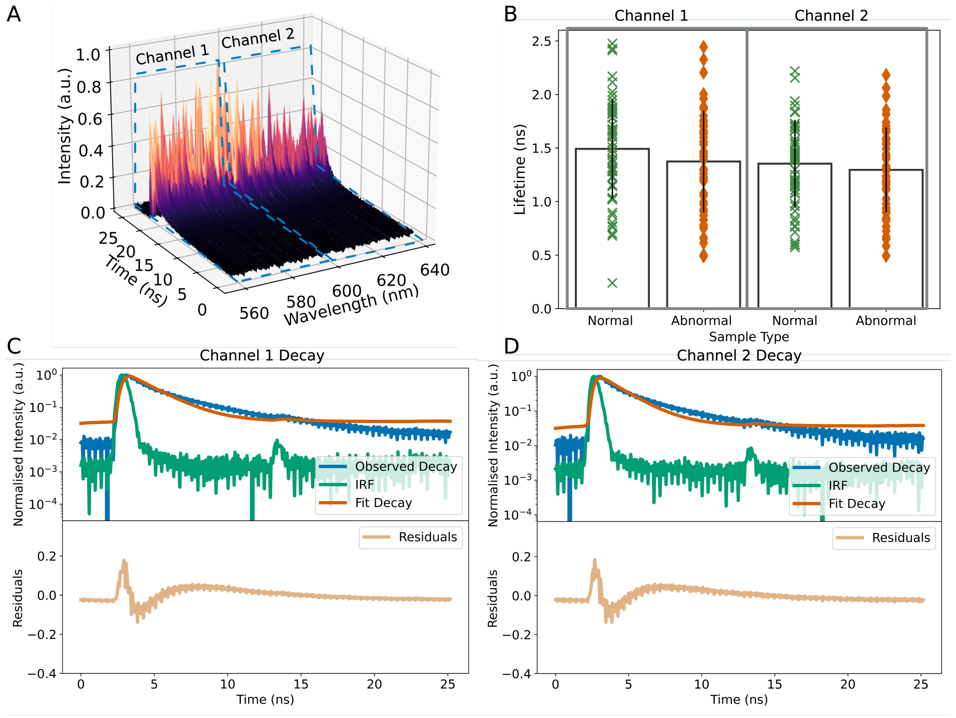

Fig. S1. The histogram and results of marginalising the histogram, splitting the observed decay traces into two spectrally distinct channels. The observed decay from the first channel is a product of summing all channels between 557.13 nm-597.42 nm across the time axis, the second channel is a product of summing all channels between 597.93 nm-638.22 nm across the time axis **A)**. The lifetime values between all normal (green crosses) and abnormal (orange diamonds) samples from both channel 1 and 2 is shown **B)**. The bar plots represent the average lifetime value from that sample type in the specified channel. The results from a sample following the least squares estimation of the fluorescence decay from the two channels is shown **C) and D)**. The blue decay represents the observed decay, the green represents the instrument response function and the orange decay represents the decay as a result of the fitted data. The subplot below the decay trace represent the residuals of between the observed and fitted data.

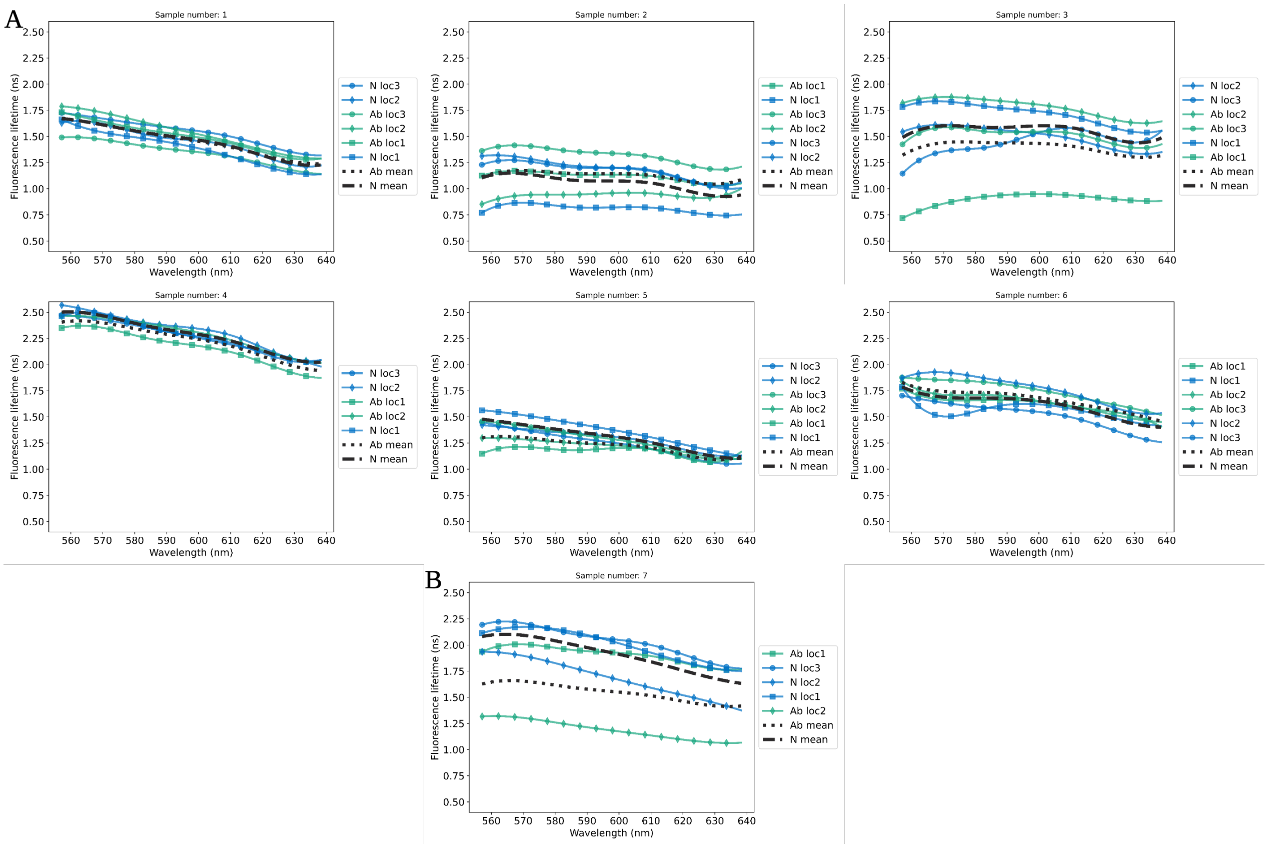

Fig. S2. Spectral fluorescence lifetime profiles of paired normal and cancer diagnosed as Adenocarcinoma. **A)** represent the 5 early stage samples and **B)** is a lung metastasis.

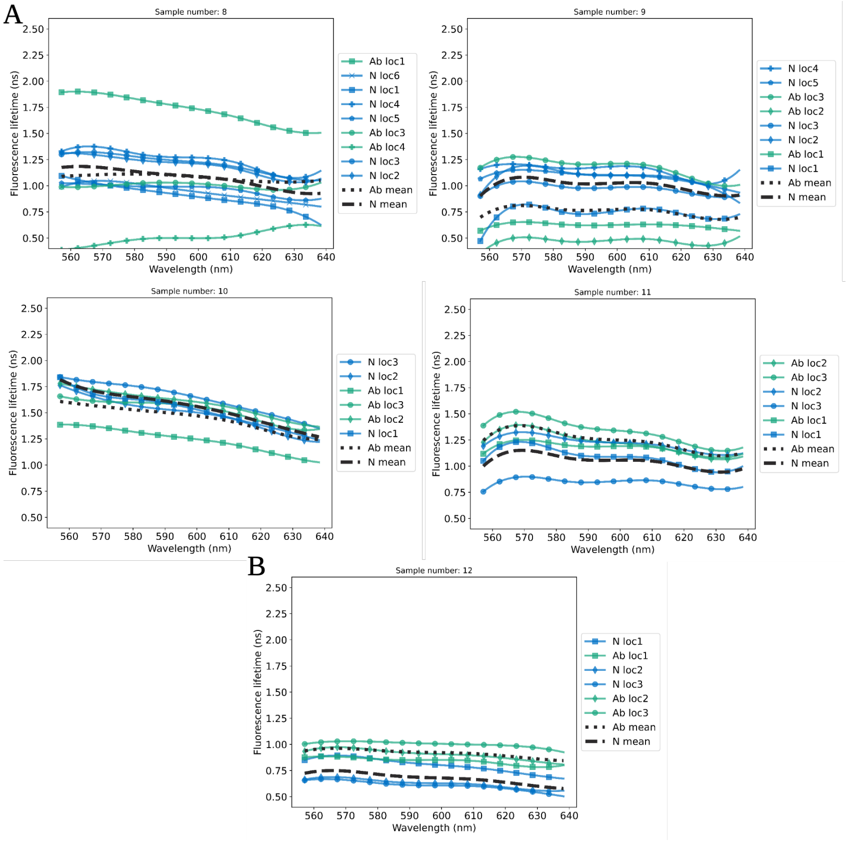

Fig. S3. Spectral fluorescence lifetime profiles of paired normal and cancer diagnosed as Squamous cell carcinoma. **A)** represent the 4 samples classified as stage 3A and **B)** is a sample classified as stage 2B.

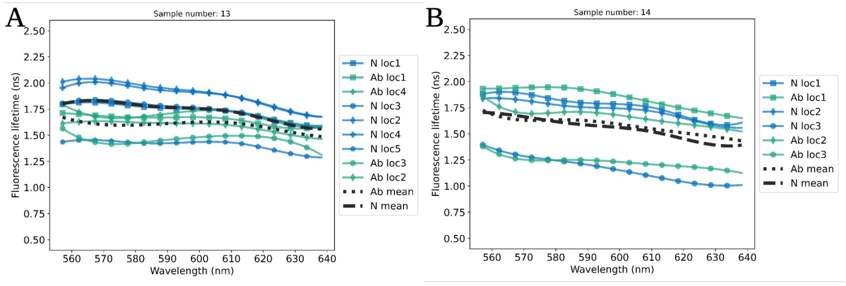

Fig. S4. Spectral fluorescence lifetime profiles of paired normal and cancer diagnosed as either large cell neuroendocrine carcinoma stage 3A **A)** or malignant melanoma **B)**

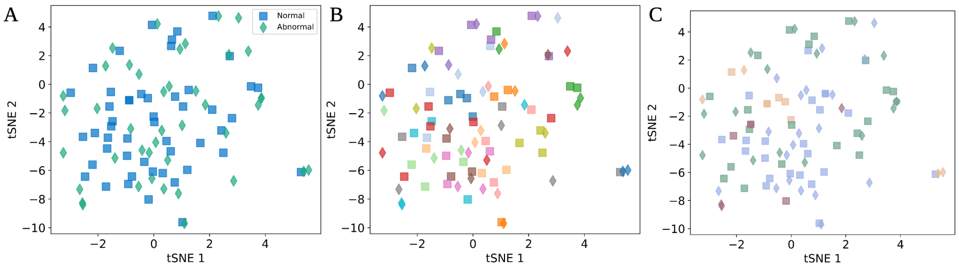

Fig. S5. tSNE plots representing the pairwise distance between the spectral fluorescence lifetime of 14 *ex vivo* clinically defined normal and abnormal samples. This distance was measured using the Procrustes similarity test. **A)** shows the difference between all normal samples (blue square) compared to the abnormal samples (green diamond). **B)** shows the difference between and within all samples, normal (square) and abnormal (diamond). **C)** shows the difference between samples clinically defined as Adenocarcinoma (blue), Squamous cell (green), Neuroendocrine (beige) and Melanoma (mauve).

REFERENCES

1. P. Uehlinger, T. Gabrecht, T. Glanzmann, *et al.*, "In vivo time-resolved spectroscopy of the human bronchial early cancer autofluorescence," J. biomedical optics **14**, 024011–024011 (2009).
2. M. Wang, F. Tang, X. Pan *et al.*, "Rapid diagnosis and intraoperative margin assessment of human lung cancer with fluorescence lifetime imaging microscopy," BBA clinical **8**, 7–13 (2017).
3. S. Fernandes, G. Williams, E. Williams, *et al.*, "Fluorescence-lifetime imaging: a novel diagnostic tool for suspected lung cancer," (2021).
4. M. W. Conklin, P. P. Provenzano, K. W. Eliceiri, *et al.*, "Fluorescence lifetime imaging of endogenous fluorophores in histopathology sections reveals differences between normal and tumor epithelium in carcinoma in situ of the breast," Cell biochemistry biophysics **53**, 145–157 (2009).
5. P. J. Tadrous, J. Siegel, P. M. French, *et al.*, "Fluorescence lifetime imaging of unstained tissues: early results in human breast cancer," The J. Pathol. A J. Pathol. Soc. Gt. Br. Irel. **199**, 309–317 (2003).
6. Y. Wang, C. Song, M. Wang, *et al.*, "Rapid, label-free, and highly sensitive detection of cervical cancer with fluorescence lifetime imaging microscopy," IEEE J. Sel. Top. Quantum Electron. **22**, 228–234 (2015).
7. M. Brandao, R. Iwakura, F. Basilio, *et al.*, "Fluorescence lifetime of normal, benign, and malignant thyroid tissues," J. biomedical optics **20**, 067003–067003 (2015).
8. J. McGinty, N. P. Galletly, C. Dunsby, *et al.*, "Wide-field fluorescence lifetime imaging of cancer," Biomed. optics express **1**, 627–640 (2010).
9. P. De Beule, C. Dunsby, N. Galletly, *et al.*, "A hyperspectral fluorescence lifetime probe for skin cancer diagnosis," Rev. scientific instruments **78**, 123101 (2007).
